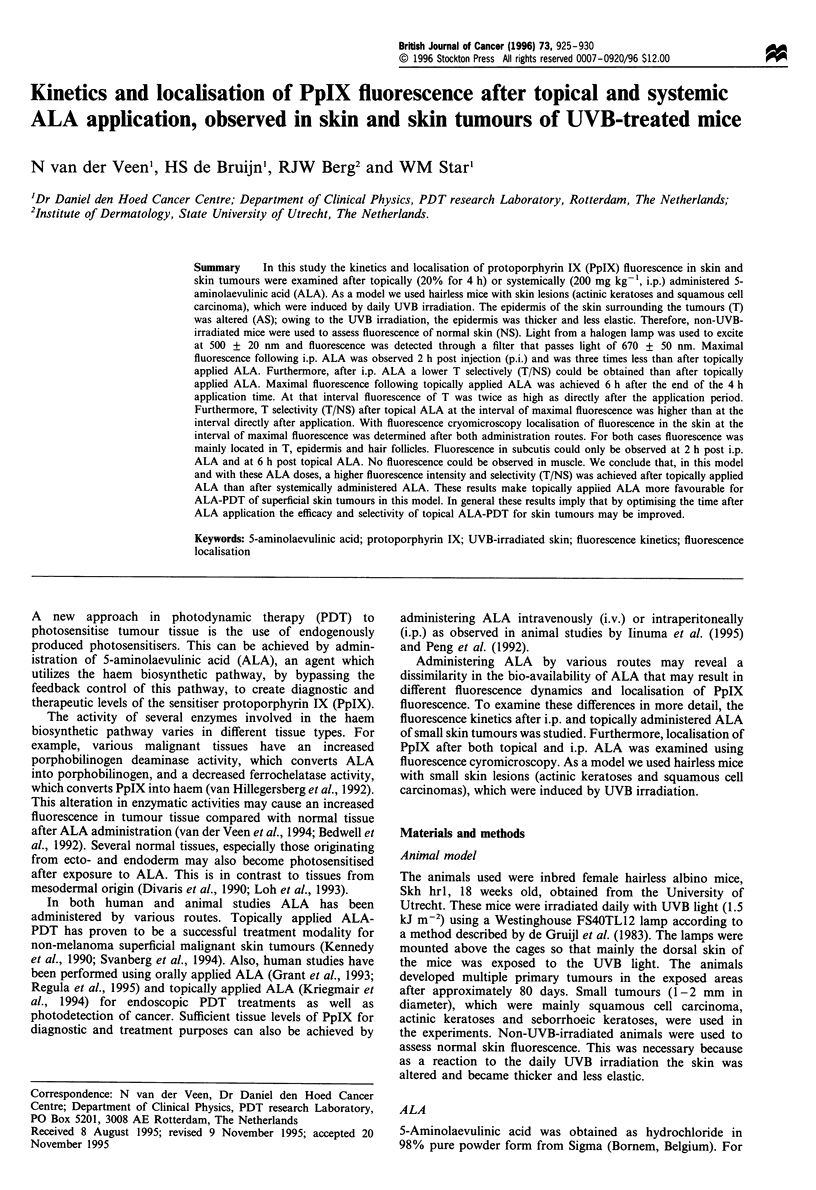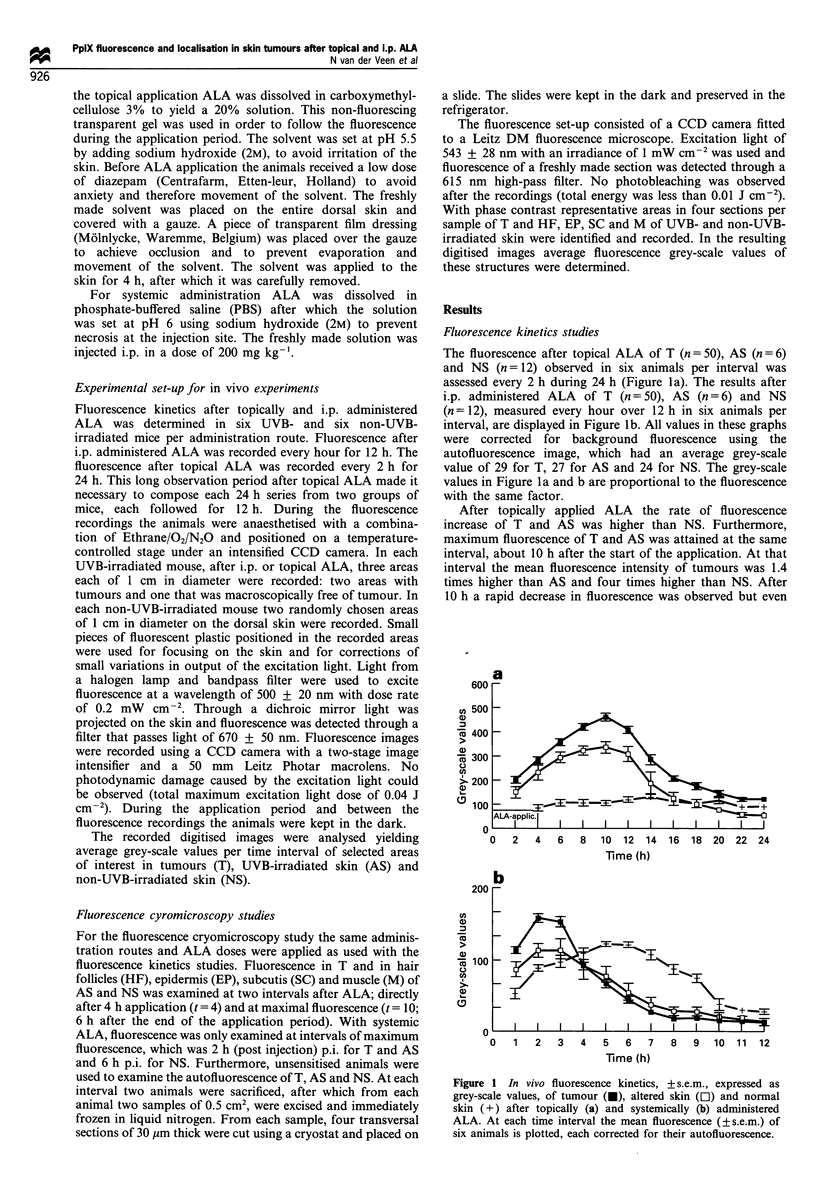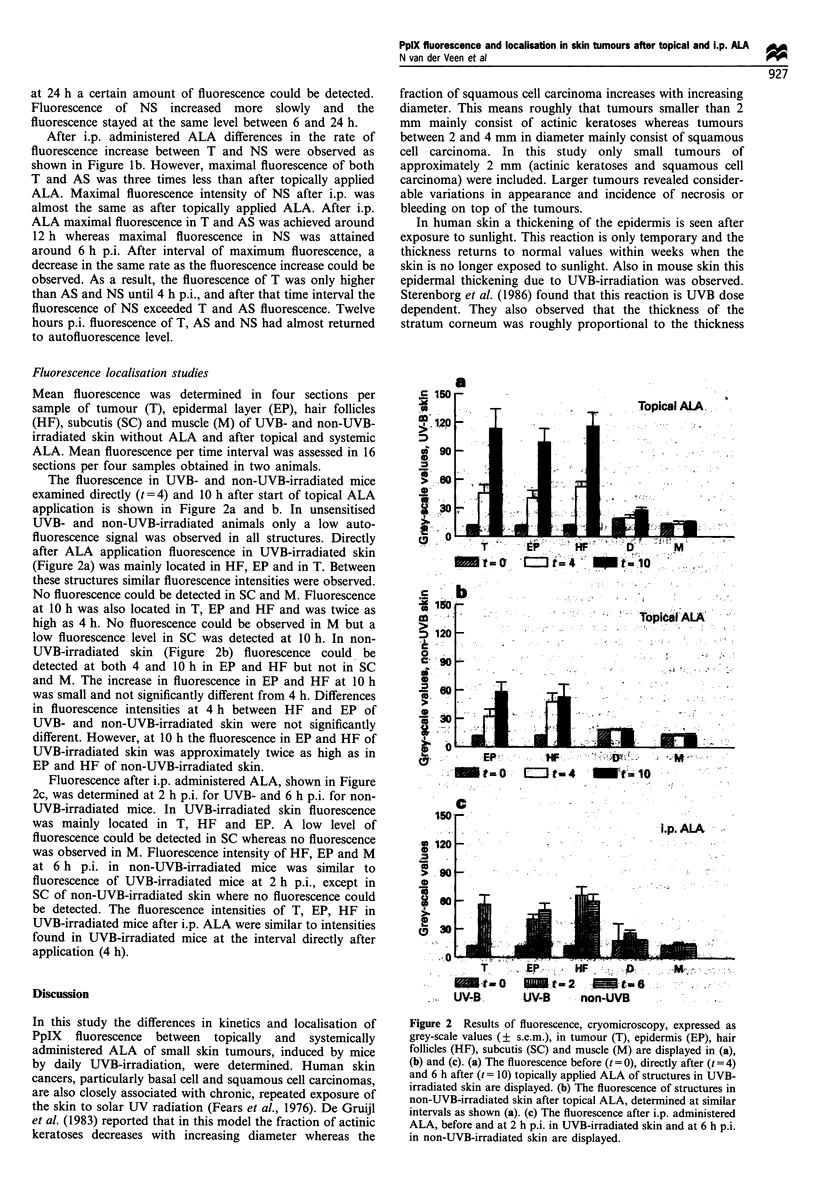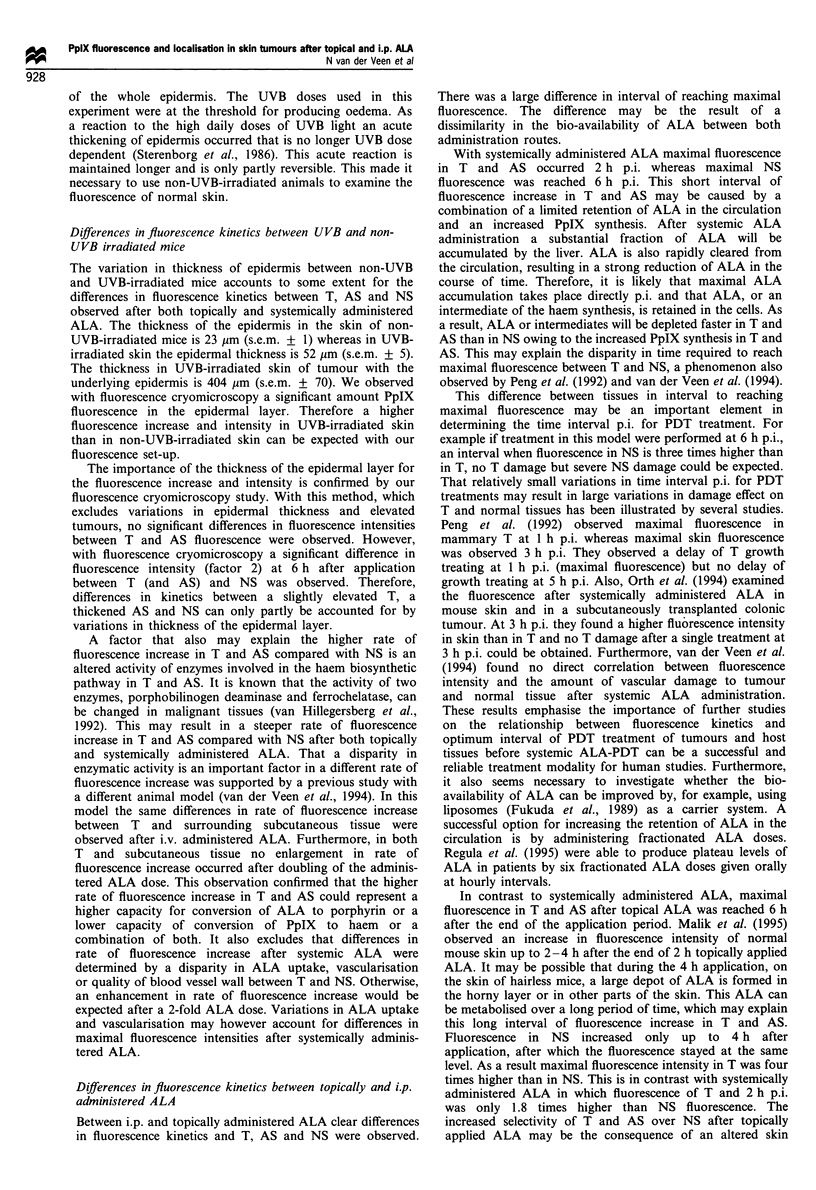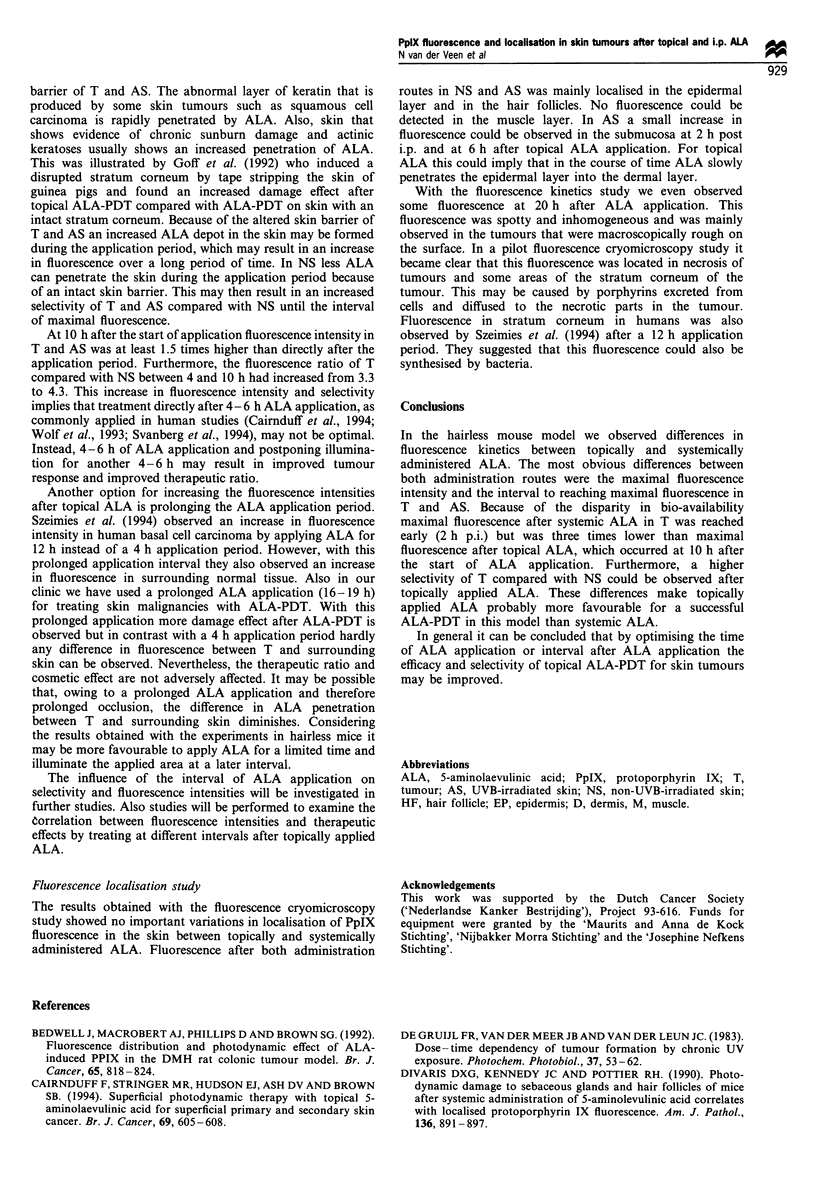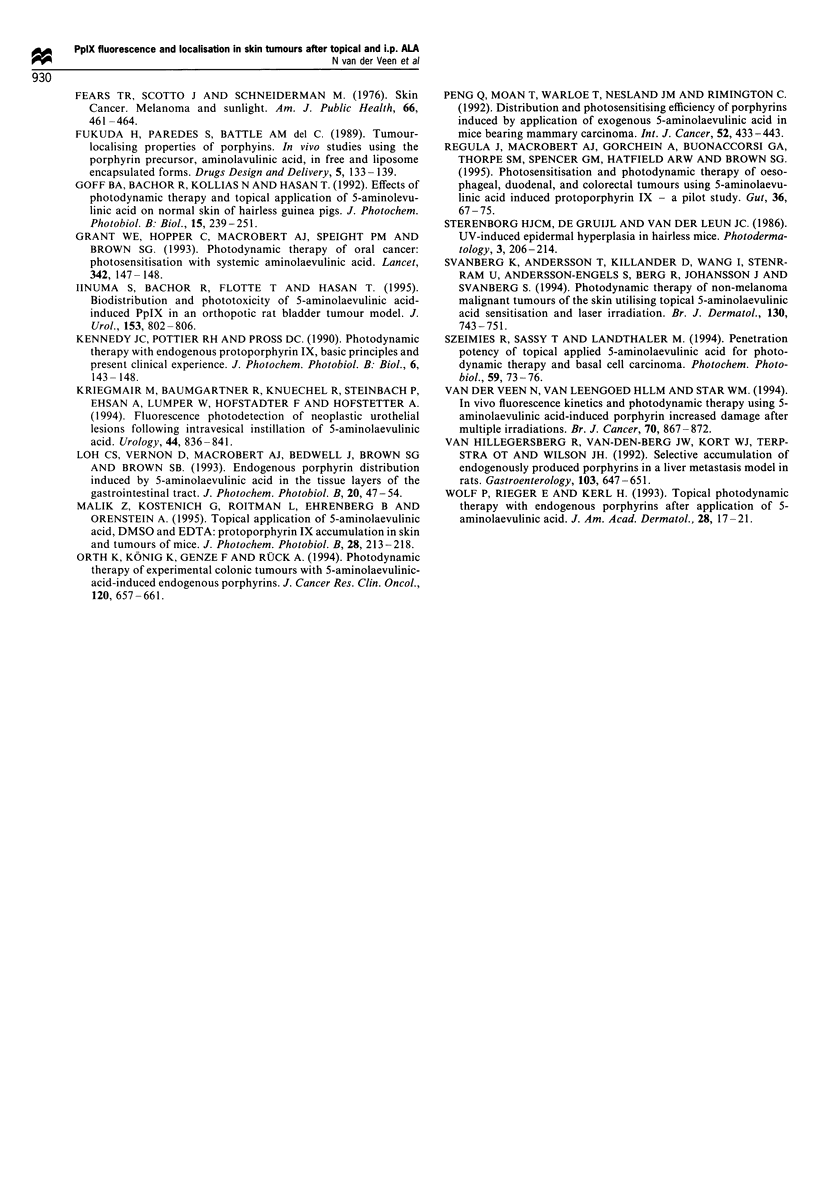# Kinetics and localisation of PpIX fluorescence after topical and systemic ALA application, observed in skin and skin tumours of UVB-treated mice.

**DOI:** 10.1038/bjc.1996.165

**Published:** 1996-04

**Authors:** N. van der Veen, H. S. de Bruijn, R. J. Berg, W. M. Star

**Affiliations:** Dr Daniel den Hoed Cancer Centre; Department of Clincal Physics, PDT research Laboratory, Rotterdam, The Netherlands.

## Abstract

In this study the kinetics and localisation of protoporphyrin IX (PpIX) fluorescence in skin and skin tumours were examined after topically (20% for 4h) or systemically (200 mg/kg,i.p.) administered 5-aminolaevulinic acid (ALA). As a model we used hairless mice with skin lesions (actinic keratoses and squamous cell carcinoma), which were induced by daily UVB irradiation. The epidermis of the skin surrounding the tumours (T) was altered (AS); owing to the UVB irradiation, the epidermis was thicker and less elastic. Therefore, non-UVB-irradiated mice were used to assess fluorescence of normal skin (NS). Light from a halogen lamp was used to excite at 500 +/- 20 nm and fluorescence was detected through a filter that passes light of 670 +/- 50 nm. Maximal fluorescence following i.p. ALA was observed 2 h post injection (p.i.) and was three times less than after topically applied ALA. Furthermore, after i.p. ALA a lower T selectively (T/NS) could be obtained than after topically applied ALA. Maximal fluorescence following topically applied ALA was achieved 6 h after the end of the 4 h application time. At that interval fluorescence of T was twice as high as directly after the application period. Furthermore, T selectivity (T/NS) after topical ALA at the interval of maximal fluorescence was higher than at the interval directly after application. With fluorescence cryomicroscopy localisation of fluorescence in the skin at the interval of maximal fluorescence was determined after both administration routes. For both cases fluorescence was mainly located in T, epidermis and hair follicles. Fluorescence in subcutis could only be observed at 2 h post i.p. ALA and a 6 h post topical ALA. No fluorescence could be observed in muscle. We conclude that, in this model and with these ALA doses, a higher fluorescence intensity and selectivity (T/NS) was achieved after topically applied ALA than after systemically administered ALA. These results make topically applied ALA more favourable for ALA-PDT of superficial skin tumours in this model. In general these results imply that by optimising the time after ALA application the efficacy and selectivity of topical ALA-PDT for skin tumours may be improved.


British Journal of Cancer (1996) 73, 925-930
© 1996 Stockton Press All rights reserved 0007-0920/96 $12.00

### Kinetics and localisation of PpIX fluorescence after topical and systemic ALA application, observed in skin and skin tumours of UVB-treated mice

N van der Veen[1], HS de Bruijn[1], RJW Berg[2] and WM Star[1]

[1]*Dr Daniel den Hoed Cancer Centre; Department of Clinical Physics, PDT research Laboratory, Rotterdam, The Netherlands;* [2]*Institute of Dermatology, State University of Utrecht, The Netherlands.*

**Summary** In this study the kinetics and localisation of protoporphyrin IX (PpIX) fluorescence in skin and skin tumours were examined after topically (20% for 4 h) or systemically (200 mg $kg^{-1}$, i.p.) administered 5-aminolaevulinic acid (ALA). As a model we used hairless mice with skin lesions (actinic keratoses and squamous cell carcinoma), which were induced by daily UVB irradiation. The epidermis of the skin surrounding the tumours (T) was altered (AS); owing to the UVB irradiation, the epidermis was thicker and less elastic. Therefore, non-UVB-irradiated mice were used to assess fluorescence of normal skin (NS). Light from a halogen lamp was used to excite at 500 $\pm$ 20 nm and fluorescence was detected through a filter that passes light of 670 $\pm$ 50 nm. Maximal fluorescence following i.p. ALA was observed 2 h post injection (p.i.) and was three times less than after topically applied ALA. Furthermore, after i.p. ALA a lower T selectively (T/NS) could be obtained than after topically applied ALA. Maximal fluorescence following topically applied ALA was achieved 6 h after the end of the 4 h application time. At that interval fluorescence of T was twice as high as directly after the application period. Furthermore, T selectivity (T/NS) after topical ALA at the interval of maximal fluorescence was higher than at the interval directly after application. With fluorescence cryomicroscopy localisation of fluorescence in the skin at the interval of maximal fluorescence was determined after both administration routes. For both cases fluorescence was mainly located in T, epidermis and hair follicles. Fluorescence in subcutis could only be observed at 2 h post i.p. ALA and at 6 h post topical ALA. No fluorescence could be observed in muscle. We conclude that, in this model and with these ALA doses, a higher fluorescence intensity and selectivity (T/NS) was achieved after topically applied ALA than after systemically administered ALA. These results make topically applied ALA more favourable for ALA-PDT of superficial skin tumours in this model. In general these results imply that by optimising the time after ALA application the efficacy and selectivity of topical ALA-PDT for skin tumours may be improved.

**Keywords:** 5-aminolaevulinic acid; protoporphyrin IX; UVB-irradiated skin; fluorescence kinetics; fluorescence localisation

A new approach in photodynamic therapy (PDT) to photosensitise tumour tissue is the use of endogenously produced photosensitisers. This can be achieved by administration of 5-aminolaevulinic acid (ALA), an agent which utilizes the haem biosynthetic pathway, by bypassing the feedback control of this pathway, to create diagnostic and therapeutic levels of the sensitiser protoporphyrin IX (PpIX).

The activity of several enzymes involved in the haem biosynthetic pathway varies in different tissue types. For example, various malignant tissues have an increased porphobilinogen deaminase activity, which converts ALA into porphobilinogen, and a decreased ferrochelatase activity, which converts PpIX into haem (van Hillegersberg *et al.*, 1992). This alteration in enzymatic activities may cause an increased fluorescence in tumour tissue compared with normal tissue after ALA administration (van der Veen *et al.*, 1994; Bedwell *et al.*, 1992). Several normal tissues, especially those originating from ecto- and endoderm may also become photosensitised after exposure to ALA. This is in contrast to tissues from mesodermal origin (Divaris *et al.*, 1990; Loh *et al.*, 1993).

In both human and animal studies ALA has been administered by various routes. Topically applied ALA-PDT has proven to be a successful treatment modality for non-melanoma superficial malignant skin tumours (Kennedy *et al.*, 1990; Svanberg *et al.*, 1994). Also, human studies have been performed using orally applied ALA (Grant *et al.*, 1993; Regula *et al.*, 1995) and topically applied ALA (Kriegmair *et al.*, 1994) for endoscopic PDT treatments as well as photodetection of cancer. Sufficient tissue levels of PpIX for diagnostic and treatment purposes can also be achieved by administering ALA intravenously (i.v.) or intraperitoneally (i.p.) as observed in animal studies by Iinuma *et al.* (1995) and Peng *et al.* (1992).

Administering ALA by various routes may reveal a dissimilarity in the bio-availability of ALA that may result in different fluorescence dynamics and localisation of PpIX fluorescence. To examine these differences in more detail, the fluorescence kinetics after i.p. and topically administered ALA of small skin tumours was studied. Furthermore, localisation of PpIX after both topical and i.p. ALA was examined using fluorescence cyromicroscopy. As a model we used hairless mice with small skin lesions (actinic keratoses and squamous cell carcinomas), which were induced by UVB irradiation.

### Materials and methods

### *Animal model*

The animals used were inbred female hairless albino mice, Skh hrl, 18 weeks old, obtained from the University of Utrecht. These mice were irradiated daily with UVB light (1.5 kJ $m^{-2}$) using a Westinghouse FS40TL12 lamp according to a method described by de Gruijl *et al.* (1983). The lamps were mounted above the cages so that mainly the dorsal skin of the mice was exposed to the UVB light. The animals developed multiple primary tumours in the exposed areas after approximately 80 days. Small tumours (1-2 mm in diameter), which were mainly squamous cell carcinoma, actinic keratoses and seborrhoeic keratoses, were used in the experiments. Non-UVB-irradiated animals were used to assess normal skin fluorescence. This was necessary because as a reaction to the daily UVB irradiation the skin was altered and became thicker and less elastic.

### *ALA*

5-Aminolaevulinic acid was obtained as hydrochloride in 98% pure powder form from Sigma (Bornem, Belgium). For

Correspondence: N van der Veen, Dr Daniel den Hoed Cancer Centre; Department of Clinical Physics, PDT research Laboratory, PO Box 5201, 3008 AE Rotterdam, The Netherlands
Received 8 August 1995; revised 9 November 1995; accepted 20 November 1995

the topical application ALA was dissolved in carboxymethylcellulose 3% to yield a 20% solution. This non-fluorescing transparent gel was used in order to follow the fluorescence during the application period. The solvent was set at pH 5.5 by adding sodium hydroxide (2M), to avoid irritation of the skin. Before ALA application the animals received a low dose of diazepam (Centrafarm, Etten-leur, Holland) to avoid anxiety and therefore movement of the solvent. The freshly made solvent was placed on the entire dorsal skin and covered with a gauze. A piece of transparent film dressing (Mölnlycke, Waremme, Belgium) was placed over the gauze to achieve occlusion and to prevent evaporation and movement of the solvent. The solvent was applied to the skin for 4 h, after which it was carefully removed.

For systemic administration ALA was dissolved in phosphate-buffered saline (PBS) after which the solution was set at pH 6 using sodium hydroxide (2M) to prevent necrosis at the injection site. The freshly made solution was injected i.p. in a dose of 200 mg $kg^{-1}$.

### *Experimental set-up for* in vivo *experiments*

Fluorescence kinetics after topically and i.p. administered ALA was determined in six UVB- and six non-UVB-irradiated mice per administration route. Fluorescence after i.p. administered ALA was recorded every hour for 12 h. The fluorescence after topical ALA was recorded every 2 h for 24 h. This long observation period after topical ALA made it necessary to compose each 24 h series from two groups of mice, each followed for 12 h. During the fluorescence recordings the animals were anaesthetised with a combination of Ethrane/$O_2$/$N_2O$ and positioned on a temperature-controlled stage under an intensified CCD camera. In each UVB-irradiated mouse, after i.p. or topical ALA, three areas each of 1 cm in diameter were recorded: two areas with tumours and one that was macroscopically free of tumour. In each non-UVB-irradiated mouse two randomly chosen areas of 1 cm in diameter on the dorsal skin were recorded. Small pieces of fluorescent plastic positioned in the recorded areas were used for focusing on the skin and for corrections of small variations in output of the excitation light. Light from a halogen lamp and bandpass filter were used to excite fluorescence at a wavelength of 500 ± 20 nm with dose rate of 0.2 mW $cm^{-2}$. Through a dichroic mirror light was projected on the skin and fluorescence was detected through a filter that passes light of 670 ± 50 nm. Fluorescence images were recorded using a CCD camera with a two-stage image intensifier and a 50 mm Leitz Photar macrolens. No photodynamic damage caused by the excitation light could be observed (total maximum excitation light dose of 0.04 J $cm^{-2}$). During the application period and between the fluorescence recordings the animals were kept in the dark.

The recorded digitised images were analysed yielding average grey-scale values per time interval of selected areas of interest in tumours (T), UVB-irradiated skin (AS) and non-UVB-irradiated skin (NS).

### *Fluorescence cyromicroscopy studies*

For the fluorescence cryomicroscopy study the same administration routes and ALA doses were applied as used with the fluorescence kinetics studies. Fluorescence in T and in hair follicles (HF), epidermis (EP), subcutis (SC) and muscle (M) of AS and NS was examined at two intervals after ALA; directly after 4 h application ($t = 4$) and at maximal fluorescence ($t = 10$; 6 h after the end of the application period). With systemic ALA, fluorescence was only examined at intervals of maximum fluorescence, which was 2 h (post injection) p.i. for T and AS and 6 h p.i. for NS. Furthermore, unsensitised animals were used to examine the autofluorescence of T, AS and NS. At each interval two animals were sacrificed, after which from each animal two samples of 0.5 $cm^2$, were excised and immediately frozen in liquid nitrogen. From each sample, four transversal sections of 30 $\mu m$ thick were cut using a cryostat and placed on a slide. The slides were kept in the dark and preserved in the refrigerator.

The fluorescence set-up consisted of a CCD camera fitted to a Leitz DM fluorescence microscope. Excitation light of 543 ± 28 nm with an irradiance of 1 mW $cm^{-2}$ was used and fluorescence of a freshly made section was detected through a 615 nm high-pass filter. No photobleaching was observed after the recordings (total energy was less than 0.01 J $cm^{-2}$). With phase contrast representative areas in four sections per sample of T and HF, EP, SC and M of UVB- and non-UVB-irradiated skin were identified and recorded. In the resulting digitised images average fluorescence grey-scale values of these structures were determined.

### Results

### *Fluorescence kinetics studies*

The fluorescence after topical ALA of T ($n = 50$), AS ($n = 6$) and NS ($n = 12$) observed in six animals per interval was assessed every 2 h during 24 h (Figure 1a). The results after i.p. administered ALA of T ($n = 50$), AS ($n = 6$) and NS ($n = 12$), measured every hour over 12 h in six animals per interval, are displayed in Figure 1b. All values in these graphs were corrected for background fluorescence using the autofluorescence image, which had an average grey-scale value of 29 for T, 27 for AS and 24 for NS. The grey-scale values in Figure 1a and b are proportional to the fluorescence with the same factor.

After topically applied ALA the rate of fluorescence increase of T and AS was higher than NS. Furthermore, maximum fluorescence of T and AS was attained at the same interval, about 10 h after the start of the application. At that interval the mean fluorescence intensity of tumours was 1.4 times higher than AS and four times higher than NS. After 10 h a rapid decrease in fluorescence was observed but even

**Figure 1** *In vivo* fluorescence kinetics, ±s.e.m., expressed as grey-scale values, of tumour (■), altered skin (□) and normal skin (+) after topically (**a**) and systemically (**b**) administered ALA. At each time interval the mean fluorescence (±s.e.m.) of six animals is plotted, each corrected for their autofluorescence.

at 24 h a certain amount of fluorescence could be detected. Fluorescence of NS increased more slowly and the fluorescence stayed at the same level between 6 and 24 h.

After i.p. administered ALA differences in the rate of fluorescence increase between T and NS were observed as shown in Figure 1b. However, maximal fluorescence of both T and AS was three times less than after topically applied ALA. Maximal fluorescence intensity of NS after i.p. was almost the same as after topically applied ALA. After i.p. ALA maximal fluorescence in T and AS was achieved around 12 h whereas maximal fluorescence in NS was attained around 6 h p.i. After interval of maximum fluorescence, a decrease in the same rate as the fluorescence increase could be observed. As a result, the fluorescence of T was only higher than AS and NS until 4 h p.i., and after that time interval the fluorescence of NS exceeded T and AS fluorescence. Twelve hours p.i. fluorescence of T, AS and NS had almost returned to autofluorescence level.

*Fluorescence localisation studies*

Mean fluorescence was determined in four sections per sample of tumour (T), epidermal layer (EP), hair follicles (HF), subcutis (SC) and muscle (M) of UVB- and non-UVB-irradiated skin without ALA and after topical and systemic ALA. Mean fluorescence per time interval was assessed in 16 sections per four samples obtained in two animals.

The fluorescence in UVB- and non-UVB-irradiated mice examined directly ($t=4$) and 10 h after start of topical ALA application is shown in Figure 2a and b. In unsensitised UVB- and non-UVB-irradiated animals only a low auto-fluorescence signal was observed in all structures. Directly after ALA application fluorescence in UVB-irradiated skin (Figure 2a) was mainly located in HF, EP and in T. Between these structures similar fluorescence intensities were observed. No fluorescence could be detected in SC and M. Fluorescence at 10 h was also located in T, EP and HF and was twice as high as 4 h. No fluorescence could be observed in M but a low fluorescence level in SC was detected at 10 h. In non-UVB-irradiated skin (Figure 2b) fluorescence could be detected at both 4 and 10 h in EP and HF but not in SC and M. The increase in fluorescence in EP and HF at 10 h was small and not significantly different from 4 h. Differences in fluorescence intensities at 4 h between HF and EP of UVB- and non-UVB-irradiated skin were not significantly different. However, at 10 h the fluorescence in EP and HF of UVB-irradiated skin was approximately twice as high as in EP and HF of non-UVB-irradiated skin.

Fluorescence after i.p. administered ALA, shown in Figure 2c, was determined at 2 h p.i. for UVB- and 6 h p.i. for non-UVB-irradiated mice. In UVB-irradiated skin fluorescence was mainly located in T, HF and EP. A low level of fluorescence could be detected in SC whereas no fluorescence was observed in M. Fluorescence intensity of HF, EP and M at 6 h p.i. in non-UVB-irradiated mice was similar to fluorescence of UVB-irradiated mice at 2 h p.i., except in SC of non-UVB-irradiated skin where no fluorescence could be detected. The fluorescence intensities of T, EP, HF in UVB-irradiated mice after i.p. ALA were similar to intensities found in UVB-irradiated mice at the interval directly after application (4 h).

### Discussion

In this study the differences in kinetics and localisation of PpIX fluorescence between topically and systemically administered ALA of small skin tumours, induced by mice by daily UVB-irradiation, were determined. Human skin cancers, particularly basal cell and squamous cell carcinomas, are also closely associated with chronic, repeated exposure of the skin to solar UV radiation (Fears *et al.*, 1976). De Gruijl *et al.* (1983) reported that in this model the fraction of actinic keratoses decreases with increasing diameter whereas the fraction of squamous cell carcinoma increases with increasing diameter. This means roughly that tumours smaller than 2 mm mainly consist of actinic keratoses whereas tumours between 2 and 4 mm in diameter mainly consist of squamous cell carcinoma. In this study only small tumours of approximately 2 mm (actinic keratoses and squamous cell carcinoma) were included. Larger tumours revealed considerable variations in appearance and incidence of necrosis or bleeding on top of the tumours.

In human skin a thickening of the epidermis is seen after exposure to sunlight. This reaction is only temporary and the thickness returns to normal values within weeks when the skin is no longer exposed to sunlight. Also in mouse skin this epidermal thickening due to UVB-irradiation was observed. Sterenborg *et al.* (1986) found that this reaction is UVB dose dependent. They also observed that the thickness of the stratum corneum was roughly proportional to the thickness

**Figure 2** Results of fluorescence, cryomicroscopy, expressed as grey-scale values (± s.e.m.), in tumour (T), epidermis (EP), hair follicles (HF), subcutis (SC) and muscle (M) are displayed in (**a**), (**b**) and (**c**). (**a**) The fluorescence before ($t=0$), directly after ($t=4$) and 6 h after ($t=10$) topically applied ALA of structures in UVB-irradiated skin are displayed. (**b**) The fluorescence of structures in non-UVB-irradiated skin after topical ALA, determined at similar intervals as shown (**a**). (**c**) The fluorescence after i.p. administered ALA, before and at 2 h p.i. in UVB-irradiated skin and at 6 h p.i. in non-UVB-irradiated skin are displayed.

of the whole epidermis. The UVB doses used in this experiment were at the threshold for producing oedema. As a reaction to the high daily doses of UVB light an acute thickening of epidermis occurred that is no longer UVB dose dependent (Sterenborg *et al.*, 1986). This acute reaction is maintained longer and is only partly reversible. This made it necessary to use non-UVB-irradiated animals to examine the fluorescence of normal skin.

*Differences in fluorescence kinetics between UVB and non-UVB irradiated mice*

The variation in thickness of epidermis between non-UVB and UVB-irradiated mice accounts to some extent for the differences in fluorescence kinetics between T, AS and NS observed after both topically and systemically administered ALA. The thickness of the epidermis in the skin of non-UVB-irradiated mice is 23 $\mu$m (s.e.m. $\pm$ 1) whereas in UVB-irradiated skin the epidermal thickness is 52 $\mu$m (s.e.m. $\pm$ 5). The thickness in UVB-irradiated skin of tumour with the underlying epidermis is 404 $\mu$m (s.e.m. $\pm$ 70). We observed with fluorescence cryomicroscopy a significant amount PpIX fluorescence in the epidermal layer. Therefore a higher fluorescence increase and intensity in UVB-irradiated skin than in non-UVB-irradiated skin can be expected with our fluorescence set-up.

The importance of the thickness of the epidermal layer for the fluorescence increase and intensity is confirmed by our fluorescence cryomicroscopy study. With this method, which excludes variations in epidermal thickness and elevated tumours, no significant differences in fluorescence intensities between T and AS fluorescence were observed. However, with fluorescence cryomicroscopy a significant difference in fluorescence intensity (factor 2) at 6 h after application between T (and AS) and NS was observed. Therefore, differences in kinetics between a slightly elevated T, a thickened AS and NS can only partly be accounted for by variations in thickness of the epidermal layer.

A factor that also may explain the higher rate of fluorescence increase in T and AS compared with NS is an altered activity of enzymes involved in the haem biosynthetic pathway in T and AS. It is known that the activity of two enzymes, porphobilinogen deaminase and ferrochelatase, can be changed in malignant tissues (van Hillegersberg *et al.*, 1992). This may result in a steeper rate of fluorescence increase in T and AS compared with NS after both topically and systemically administered ALA. That a disparity in enzymatic activity is an important factor in a different rate of fluorescence increase was supported by a previous study with a different animal model (van der Veen *et al.*, 1994). In this model the same differences in rate of fluorescence increase between T and surrounding subcutaneous tissue were observed after i.v. administered ALA. Furthermore, in both T and subcutaneous tissue no enlargement in rate of fluorescence increase occurred after doubling of the administered ALA dose. This observation confirmed that the higher rate of fluorescence increase in T and AS could represent a higher capacity for conversion of ALA to porphyrin or a lower capacity of conversion of PpIX to haem or a combination of both. It also excludes that differences in rate of fluorescence increase after systemic ALA were determined by a disparity in ALA uptake, vascularisation or quality of blood vessel wall between T and NS. Otherwise, an enhancement in rate of fluorescence increase would be expected after a 2-fold ALA dose. Variations in ALA uptake and vascularisation may however account for differences in maximal fluorescence intensities after systemically administered ALA.

*Differences in fluorescence kinetics between topically and i.p. administered ALA*

Between i.p. and topically administered ALA clear differences in fluorescence kinetics and T, AS and NS were observed. There was a large difference in interval of reaching maximal fluorescence. The difference may be the result of a dissimilarity in the bio-availability of ALA between both administration routes.

With systemically administered ALA maximal fluorescence in T and AS occurred 2 h p.i. whereas maximal NS fluorescence was reached 6 h p.i. This short interval of fluorescence increase in T and AS may be caused by a combination of a limited retention of ALA in the circulation and an increased PpIX synthesis. After systemic ALA administration a substantial fraction of ALA will be accumulated by the liver. ALA is also rapidly cleared from the circulation, resulting in a strong reduction of ALA in the course of time. Therefore, it is likely that maximal ALA accumulation takes place directly p.i. and that ALA, or an intermediate of the haem synthesis, is retained in the cells. As a result, ALA or intermediates will be depleted faster in T and AS than in NS owing to the increased PpIX synthesis in T and AS. This may explain the disparity in time required to reach maximal fluorescence between T and NS, a phenomenon also observed by Peng *et al.* (1992) and van der Veen *et al.* (1994).

This difference between tissues in interval to reaching maximal fluorescence may be an important element in determining the time interval p.i. for PDT treatment. For example if treatment in this model were performed at 6 h p.i., an interval when fluorescence in NS is three times higher than in T, no T damage but severe NS damage could be expected. That relatively small variations in time interval p.i. for PDT treatments may result in large variations in damage effect on T and normal tissues has been illustrated by several studies. Peng *et al.* (1992) observed maximal fluorescence in mammary T at 1 h p.i. whereas maximal skin fluorescence was observed 3 h p.i. They observed a delay of T growth treating at 1 h p.i. (maximal fluorescence) but no delay of growth treating at 5 h p.i. Also, Orth *et al.* (1994) examined the fluorescence after systemically administered ALA in mouse skin and in a subcutaneously transplanted colonic tumour. At 3 h p.i. they found a higher fluorescence intensity in skin than in T and no T damage after a single treatment at 3 h p.i. could be obtained. Furthermore, van der Veen *et al.* (1994) found no direct correlation between fluorescence intensity and the amount of vascular damage to tumour and normal tissue after systemic ALA administration. These results emphasise the importance of further studies on the relationship between fluorescence kinetics and optimum interval of PDT treatment of tumours and host tissues before systemic ALA-PDT can be a successful and reliable treatment modality for human studies. Furthermore, it also seems necessary to investigate whether the bio-availability of ALA can be improved by, for example, using liposomes (Fukuda *et al.*, 1989) as a carrier system. A successful option for increasing the retention of ALA in the circulation is by administering fractionated ALA doses. Regula *et al.* (1995) were able to produce plateau levels of ALA in patients by six fractionated ALA doses given orally at hourly intervals.

In contrast to systemically administered ALA, maximal fluorescence in T and AS after topical ALA was reached 6 h after the end of the application period. Malik *et al.* (1995) observed an increase in fluorescence intensity of normal mouse skin up to 2–4 h after the end of 2 h topically applied ALA. It may be possible that during the 4 h application, on the skin of hairless mice, a large depot of ALA is formed in the horny layer or in other parts of the skin. This ALA can be metabolised over a long period of time, which may explain this long interval of fluorescence increase in T and AS. Fluorescence in NS increased only up to 4 h after application, after which the fluorescence stayed at the same level. As a result maximal fluorescence intensity in T was four times higher than in NS. This is in contrast with systemically administered ALA in which fluorescence of T and 2 h p.i. was only 1.8 times higher than NS fluorescence. The increased selectivity of T and AS over NS after topically applied ALA may be the consequence of an altered skin barrier of T and AS. The abnormal layer of keratin that is produced by some skin tumours such as squamous cell carcinoma is rapidly penetrated by ALA. Also, skin that shows evidence of chronic sunburn damage and actinic keratoses usually shows an increased penetration of ALA. This was illustrated by Goff *et al.* (1992) who induced a disrupted stratum corneum by tape stripping the skin of guinea pigs and found an increased damage effect after topical ALA-PDT compared with ALA-PDT on skin with an intact stratum corneum. Because of the altered skin barrier of T and AS an increased ALA depot in the skin may be formed during the application period, which may result in an increase in fluorescence over a long period of time. In NS less ALA can penetrate the skin during the application period because of an intact skin barrier. This may then result in an increased selectivity of T and AS compared with NS until the interval of maximal fluorescence.

At 10 h after the start of application fluorescence intensity in T and AS was at least 1.5 times higher than directly after the application period. Furthermore, the fluorescence ratio of T compared with NS between 4 and 10 h had increased from 3.3 to 4.3. This increase in fluorescence intensity and selectivity implies that treatment directly after 4–6 h ALA application, as commonly applied in human studies (Cairnduff *et al.*, 1994; Wolf *et al.*, 1993; Svanberg *et al.*, 1994), may not be optimal. Instead, 4–6 h of ALA application and postponing illumination for another 4–6 h may result in improved tumour response and improved therapeutic ratio.

Another option for increasing the fluorescence intensities after topical ALA is prolonging the ALA application period. Szeimies *et al.* (1994) observed an increase in fluorescence intensity in human basal cell carcinoma by applying ALA for 12 h instead of a 4 h application period. However, with this prolonged application interval they also observed an increase in fluorescence in surrounding normal tissue. Also in our clinic we have used a prolonged ALA application (16–19 h) for treating skin malignancies with ALA-PDT. With this prolonged application more damage effect after ALA-PDT is observed but in contrast with a 4 h application period hardly any difference in fluorescence between T and surrounding skin can be observed. Nevertheless, the therapeutic ratio and cosmetic effect are not adversely affected. It may be possible that, owing to a prolonged ALA application and therefore prolonged occlusion, the difference in ALA penetration between T and surrounding skin diminishes. Considering the results obtained with the experiments in hairless mice it may be more favourable to apply ALA for a limited time and illuminate the applied area at a later interval.

The influence of the interval of ALA application on selectivity and fluorescence intensities will be investigated in further studies. Also studies will be performed to examine the correlation between fluorescence intensities and therapeutic effects by treating at different intervals after topically applied ALA.

*Fluorescence localisation study*

The results obtained with the fluorescence cryomicroscopy study showed no important variations in localisation of PpIX fluorescence in the skin between topically and systemically administered ALA. Fluorescence after both administration routes in NS and AS was mainly localised in the epidermal layer and in the hair follicles. No fluorescence could be detected in the muscle layer. In AS a small increase in fluorescence could be observed in the submucosa at 2 h post i.p. and at 6 h after topical ALA application. For topical ALA this could imply that in the course of time ALA slowly penetrates the epidermal layer into the dermal layer.

With the fluorescence kinetics study we even observed some fluorescence at 20 h after ALA application. This fluorescence was spotty and inhomogeneous and was mainly observed in the tumours that were macroscopically rough on the surface. In a pilot fluorescence cryomicroscopy study it became clear that this fluorescence was located in necrosis of tumours and some areas of the stratum corneum of the tumour. This may be caused by porphyrins excreted from cells and diffused to the necrotic parts in the tumour. Fluorescence in stratum corneum in humans was also observed by Szeimies *et al.* (1994) after a 12 h application period. They suggested that this fluorescence could also be synthesised by bacteria.

### Conclusions

In the hairless mouse model we observed differences in fluorescence kinetics between topically and systemically administered ALA. The most obvious differences between both administration routes were the maximal fluorescence intensity and the interval to reaching maximal fluorescence in T and AS. Because of the disparity in bio-availability maximal fluorescence after systemic ALA in T was reached early (2 h p.i.) but was three times lower than maximal fluorescence after topical ALA, which occurred at 10 h after the start of ALA application. Furthermore, a higher selectivity of T compared with NS could be observed after topically applied ALA. These differences make topically applied ALA probably more favourable for a successful ALA-PDT in this model than systemic ALA.

In general it can be concluded that by optimising the time of ALA application or interval after ALA application the efficacy and selectivity of topical ALA-PDT for skin tumours may be improved.

**Abbreviations**

ALA, 5-aminolaevulinic acid; PpIX, protoporphyrin IX; T, tumour; AS, UVB-irradiated skin; NS, non-UVB-irradiated skin; HF, hair follicle; EP, epidermis; D, dermis, M, muscle.

**Acknowledgements**

This work was supported by the Dutch Cancer Society ('Nederlandse Kanker Bestrijding'), Project 93-616. Funds for equipment were granted by the 'Maurits and Anna de Kock Stichting', 'Nijbakker Morra Stichting' and the 'Josephine Nefkens Stichting'.

### References

BEDWELL J, MACROBERT AJ, PHILLIPS D AND BROWN SG. (1992). Fluorescence distribution and photodynamic effect of ALA-induced PPIX in the DMH rat colonic tumour model. *Br. J. Cancer*, **65**, 818–824.

CAIRNDUFF F, STRINGER MR, HUDSON EJ, ASH DV AND BROWN SB. (1994). Superficial photodynamic therapy with topical 5-aminolaevulinic acid for superficial primary and secondary skin cancer. *Br. J. Cancer*, **69**, 605–608.

DE GRUIJL FR, VAN DER MEER JB AND VAN DER LEUN JC. (1983). Dose–time dependency of tumour formation by chronic UV exposure. *Photochem. Photobiol.*, **37**, 53–62.

DIVARIS DXG, KENNEDY JC AND POTTIER RH. (1990). Photodynamic damage to sebaceous glands and hair follicles of mice after systemic administration of 5-aminolevulinic acid correlates with localised protoporphyrin IX fluorescence. *Am. J. Pathol.*, **136**, 891–897.

FEARS TR, SCOTTO J AND SCHNEIDERMAN M. (1976). Skin Cancer. Melanoma and sunlight. *Am. J. Public Health*, **66**, 461-464.

FUKUDA H, PAREDES S, BATTLE AM del C. (1989). Tumour-localising properties of porphyins. *In vivo* studies using the porphyrin precursor, aminolavulinic acid, in free and liposome encapsulated forms. *Drugs Design and Delivery*, **5**, 133-139.

GOFF BA, BACHOR R, KOLLIAS N AND HASAN T. (1992). Effects of photodynamic therapy and topical application of 5-aminolevulinic acid on normal skin of hairless guinea pigs. *J. Photochem. Photobiol. B: Biol.*, **15**, 239-251.

GRANT WE, HOPPER C, MACROBERT AJ, SPEIGHT PM AND BROWN SG. (1993). Photodynamic therapy of oral cancer: photosensitisation with systemic aminolaevulinic acid. *Lancet*, **342**, 147-148.

IINUMA S, BACHOR R, FLOTTE T AND HASAN T. (1995). Biodistribution and phototoxicity of 5-aminolaevulinic acid-induced PpIX in an orthopotic rat bladder tumour model. *J. Urol.*, **153**, 802-806.

KENNEDY JC, POTTIER RH AND PROSS DC. (1990). Photodynamic therapy with endogenous protoporphyrin IX, basic principles and present clinical experience. *J. Photochem. Photobiol. B: Biol.*, **6**, 143-148.

KRIEGMAIR M, BAUMGARTNER R, KNUECHEL R, STEINBACH P, EHSAN A, LUMPER W, HOFSTADTER F AND HOFSTETTER A. (1994). Fluorescence photodetection of neoplastic urothelial lesions following intravesical instillation of 5-aminolaevulinic acid. *Urology*, **44**, 836-841.

LOH CS, VERNON D, MACROBERT AJ, BEDWELL J, BROWN SG AND BROWN SB. (1993). Endogenous porphyrin distribution induced by 5-aminolaevulinic acid in the tissue layers of the gastrointestinal tract. *J. Photochem. Photobiol. B*, **20**, 47-54.

MALIK Z, KOSTENICH G, ROITMAN L, EHRENBERG B AND ORENSTEIN A. (1995). Topical application of 5-aminolaevulinic acid, DMSO and EDTA: protoporphyrin IX accumulation in skin and tumours of mice. *J. Photochem. Photobiol. B*, **28**, 213-218.

ORTH K, KÖNIG K, GENZE F AND RÜCK A. (1994). Photodynamic therapy of experimental colonic tumours with 5-aminolaevulinic-acid-induced endogenous porphyrins. *J. Cancer Res. Clin. Oncol.*, **120**, 657-661.

PENG Q, MOAN T, WARLOE T, NESLAND JM AND RIMINGTON C. (1992). Distribution and photosensitising efficiency of porphyrins induced by application of exogenous 5-aminolaevulinic acid in mice bearing mammary carcinoma. *Int. J. Cancer*, **52**, 433-443.

REGULA J, MACROBERT AJ, GORCHEIN A, BUONACCORSI GA, THORPE SM, SPENCER GM, HATFIELD ARW AND BROWN SG. (1995). Photosensitisation and photodynamic therapy of oesophageal, duodenal, and colorectal tumours using 5-aminolaevulinic acid induced protoporphyrin IX – a pilot study. *Gut*, **36**, 67-75.

STERENBORG HJCM, DE GRUIJL AND VAN DER LEUN JC. (1986). UV-induced epidermal hyperplasia in hairless mice. *Photodermatology*, **3**, 206-214.

SVANBERG K, ANDERSSON T, KILLANDER D, WANG I, STENRAM U, ANDERSSON-ENGELS S, BERG R, JOHANSSON J AND SVANBERG S. (1994). Photodynamic therapy of non-melanoma malignant tumours of the skin utilising topical 5-aminolaevulinic acid sensitisation and laser irradiation. *Br. J. Dermatol.*, **130**, 743-751.

SZEIMIES R, SASSY T AND LANDTHALER M. (1994). Penetration potency of topical applied 5-aminolaevulinic acid for photodynamic therapy and basal cell carcinoma. *Photochem. Photobiol.*, **59**, 73-76.

VAN DER VEEN N, VAN LEENGOED HLLM AND STAR WM. (1994). In vivo fluorescence kinetics and photodynamic therapy using 5-aminolaevulinic acid-induced porphyrin increased damage after multiple irradiations. *Br. J. Cancer*, **70**, 867-872.

VAN HILLEGERSBERG R, VAN-DEN-BERG JW, KORT WJ, TERPSTRA OT AND WILSON JH. (1992). Selective accumulation of endogenously produced porphyrins in a liver metastasis model in rats. *Gastroenterology*, **103**, 647-651.

WOLF P, RIEGER E AND KERL H. (1993). Topical photodynamic therapy with endogenous porphyrins after application of 5-aminolaevulinic acid. *J. Am. Acad. Dermatol.*, **28**, 17-21.